# The Neuroscience Experiments System (NES)–A Software Tool to Manage Experimental Data and Its Provenance

**DOI:** 10.3389/fninf.2021.768615

**Published:** 2022-01-07

**Authors:** Margarita Ruiz-Olazar, Evandro Santos Rocha, Claudia D. Vargas, Kelly Rosa Braghetto

**Affiliations:** ^1^Research, Innovation and Dissemination Center for Neuromathematics, University of São Paulo, São Paulo, Brazil; ^2^Polytechnic Faculty, National University of Asunción, Asunción, Paraguay; ^3^Institute of Biophysics Carlos Chagas Filho, Federal University of Rio de Janeiro, Rio de Janeiro, Brazil; ^4^Department of Computer Science, Institute of Mathematics and Statistics, University of São Paulo, São Paulo, Brazil

**Keywords:** neuroscience, experiment data, data management, data provenance, open-source software

## Abstract

Computational tools can transform the manner by which neuroscientists perform their experiments. More than helping researchers to manage the complexity of experimental data, these tools can increase the value of experiments by enabling reproducibility and supporting the sharing and reuse of data. Despite the remarkable advances made in the Neuroinformatics field in recent years, there is still a lack of open-source computational tools to cope with the heterogeneity and volume of neuroscientific data and the related metadata that needs to be collected during an experiment and stored for posterior analysis. In this work, we present the Neuroscience Experiments System (NES), a free software to assist researchers in data collecting routines of clinical, electrophysiological, and behavioral experiments. NES enables researchers to efficiently perform the management of their experimental data in a secure and user-friendly environment, providing a unified repository for the experimental data of an entire research group. Furthermore, its modular software architecture is aligned with several initiatives of the neuroscience community and promotes standardized data formats for experiments and analysis reporting.

## 1. Introduction

Although the overlap between neuroscience and informatics has been growing rapidly in the recent years, collection and organization of experimental data are still frequently done manually. A neuroscience experiment may involve the generation and manipulation of large amounts of both raw and processed data. There is a wide variability in the types of data that are collected, from the form and behavior of individual neurons to measures of brain functioning. This large quantity and variety of information requires a type of database that is especially designed for this purpose. However, the provenance information of raw data is too often lost or, when digitized, ends up as text files or spread-sheets without a standardized structure (Koslow, 2000). Within this context, the reproducibility of experiments—a core scientific principle—and the reuse of data may be seriously compromised. Efforts to develop best practices must be made on four foundational principles—Findability, Accessibility, Interoperability and Reusability (FAIR), as described by Wilkinson et al. in the FAIR Guiding Principles for scientific data management and stewardship (Abrams et al., 2021).

This scenario calls for the use of computational tools to document each step of an experiment and to facilitate the electronic data capture. Although some tools have been developed and applied for this purpose (Mouček et al., 2014; Sobolev et al., 2014), there is still a lack of user-friendly software platforms that researchers can use to register different types of experiments and their working environment in a unified repository. These platforms should allow scientists to examine the data and metadata and know exactly how these were obtained, as well as how the experiment was performed. Such tools should be as easy to use as possible to reduce the time spent documenting experiments, while being able to support a wide variety of experimental designs (Peirce, 2009). Moreover, it should be platform-independent and a free/libre open-source software (FLOSS).

Addressing this problem, the Research, Innovation and Dissemination Center for Neuromathematics (NeuroMat), hosted by the University of São Paulo Research, Brazil^1^ has developed the Neuroscience Experiments System (NES), a FLOSS that assists neuroscientists in the management of experimental data while providing provenance recording and interoperability. NES is a Web system that offers a user-friendly interface, allowing quick learning. Its data model combines several proposals from the scientific community for neuroscience data and metadata representation.

The NES modular structure provides functionalities for the registration of participant data and for experiment management. The participant registration functionality allows the collection and storage of personal and social-demographic data and medical evaluations. The experiment management includes experimental protocol registration (e.g., definition of tasks, stimuli, instructions, and configuration of equipment) and electrophysiological data and metadata storage. Presently, NES is equipped with modules allowing data collection from experiments performed in humans involving electroencephalography (EEG), electromyography (EMG), transcranial magnetic stimulation (TMS), and response times.

This article presents the approach used in the NES to manage data and metadata of neuroscience experiments. The NES innovative data model was designed to provide support for a wide range of experimental designs and to allow the efficient management of all steps of the experimental protocol and their different types of data.

The remainder of this article is organized as follows. Section 2.1 provides a brief characterization of the experimental data used in neuroscience, while section 2.2 analyzes some software tools for management of this kind of data. Section 2.3 describes the NES data model and the main functionalities it supports. Section 3 presents the NES software architecture and details about its implementation. It also presents an example of use of NES in the creation of an open database. Finally, the concluding remarks, including a discussion of the limitations of NES and future directions, are presented in section 4.

## 2. Methods

### 2.1. Experimental Data in Neuroscience: Characterization

Designing an experiment includes a number of stages where the parameters and structure of the experiment are made clear. There are different types of neuroscience experiments (e.g., behavioral, cognitive, electrophysiological, and neuroimaging) with a great variability of experimental processes and a high heterogeneity of formats of collected data. An experimental process can be understood as comprising an experimental design and an experimental protocol. An experimental design includes the overall set-up of the experiment, in so far as it specifies the experimental context (e.g., how and where objects are to be arranged) and the materials and methods to be used (e.g., equipment settings). The experimental protocol is the set of step-by-step instructions that an investigator follows each time he or she runs an experiment (Sullivan, 2009). It includes a group of participants who will take part in the experiment. The steps of the experimental protocol can be performed sequentially or in parallel. After the approval of the experiment design and the experimental protocol, the group of participants is selected and the data collection starts.

The great heterogeneity of data collected in neuroscience experiments (e.g., EEG, EMG, fMRI, questionnaire responses) makes collaboration between members of the community difficult, since research groups would have to make a significant effort to standardize their lexicons and their data before collaboration could add value to such joint efforts (Hall et al., 2012). Furthermore, the information concerning the experimental process is too often lost or when digitized, it ends up becoming text files or spread-sheets without a standardized structure, or poor quality data with insufficient documentation. Sometimes, the data lacks metadata, standardized representation, or a legible structure (Barkhof, 2012).

To enhance the reproducibility of neuroscience studies, researchers need to know the precise acquisition parameters, the experimental conditions, and how the raw data were acquired. These different types of information, generally called *provenance information*, are metadata which is used to record the experimental process, the purpose of the experiment and details about its data results, as well as formal annotations and notes made by scientists.

A unified data model for handling metadata is still an open research problem. The problem is compounded when the volume of collected data begins to grow. Unlike the progress in workflow-based systems, which provide consistent mechanisms to manage the provenance of derived data generated through scientific workflows, the availability of open data models and free software tools to support raw data routine collection is limited. Thus, the creation of standardized models and formats for representing and storing raw data and its provenance information is not a trivial task and depends on collaborative efforts from the neuroscience community (Ruiz-Olazar et al., 2016).

Due to the great variability in experimental processes and the heterogeneity of collected data formats, neuroscience experimental raw data and information relating to provenance require specific and innovative ways of representation and storage. The guidelines of Gibson et al. (2008), Poldrack et al. (2008), and Frishkoff et al. (2011) include information that is considered important for data analysis and for understanding the experiment performed. However, these guidelines are neither complete data representation models nor data storage models.

### 2.2. Related Software Tools

A brief review of the open-source software tools created to support the management of neuroscience experiments shows that most of these systems can be divided in two groups: (i) those that focus on the storing and sharing of electrophysiological data and (ii) those that focus on the management of experimental protocols. Some of those that are in the first group provide interfaces to manipulate electrophysiological data objects, such as data arrays, events, regions of interest, etc., or extensively annotate these specific data objects. Those in the second group provide the management of the experimental protocol, accurate presentation of stimuli, and mechanisms for the collection of participant responses. Software tools from the two groups can be combined in order to help researchers in their data collecting routine throughout a neuroscience experiment. Table 1 compares some software tools for neuroscience experiment management.

**Table 1:** Software tools for neuroscience experiments management.

**System**	**Focus**	**Ontology**	**Open Source**
^a^EEGBase	EEG/ERP	OEN	Yes
^b^G-node	Neurophysiology	odML	Yes
^c^Psychopy	Experimental protocol	No	Yes
^d^Expyrement	Experimental protocol	No	Yes
^e^OpenSesame	Experimental protocol	No	No

a *https://eegdatabase.kiv.zcu.cz/*;

b *http://www.g-node.org/*;

c *http://www.psychopy.org/*;

d *http://www.expyriment.org/*;

e *http://osdoc.cogsci.nl/*.

Among the software tools that are in the first group is EEGBase (Mouček et al., 2014), which was designed to enable data exchange based on files. The EEGbase is a system for storage and management of EEG/ERP (electroencephalography, event-related potentials) resources, such as data, metadata, analysis tools, and documents related to experiments in respect of EEG/ERP. It provides the possibility to work offline by using a client-server approach, and data and metadata can be registered using a tablet or mobile phone based on a client-mobile system. These platforms can synchronize data with the EEGBase Web-based portal. Through this portal, researchers can store, manage, search, and share EEG/ERP data. The data and metadata are implemented according to a defined ontology and registered using predefined HTML forms. However, the metadata is registered in textual mode.

The German Neuroinformatics Node, G-node (Sobolev et al., 2014), provides a data management system with interfaces to operate with electrophysiology raw data objects. G-node is a data platform and Python library that implements tools, standards, and conventions established in an electrophysiological context. This approach is based on combining a standardized data model, NEO (Garcia et al., 2014), with a flexible and extensible metadata format, odML (Grewe et al., 2011). OdML uses the open metadata Markup Language to annotate data with information about the stimulus, data acquisition, and experimental conditions. In contrast, its extensible “key-value pairs” format does not specify the relevant information that should be registered, but it depends on the experimenter. NEO provides a flexible method of manipulating neurophysiological data and its I/O library can read a wide range of neurophysiological file formats. However, it cannot currently read relevant information such as the number of used channels, sample rate, frequency, etc. In addition, G-node offers integration with other Python tools that use these data models. However, these data models focus on cellular and intra-cellular experiments, without providing a comprehensible data schema to represent electrophysiological data such as EEG and EMG results.

Among the software tools that allow management of the experimental protocol, accurate presentation of stimuli, and collection of participant responses are Psychopy (Peirce, 2007) and Expyrement (Krause and Lindemann, 2014). Both provide an open-source software library that allows a very range of visual and auditory stimuli and a great variety of experimental designs to be generated within a framework based on Python. Expyrement aims at designing and conducting behavioral and neuroimaging experiments. Nevertheless, they do not offer a graphical interface that many users have come to expect. These packages require some effort from the users in respect of writing scripts in standard Python syntax to generate a variety of behavioral experiments.

Another related tool is OpenSesame (Mathôt et al., 2012), which provides a graphical and scripting interface to create a wide range of experiments, including psychophysical experiments, speed response time tasks, eye-tracking studies, and questionnaires. Some kinds of tasks need to be defined using Python scripting, since the tool does not provide a good graphical interface to support their definition.

The software packages described above focus on specific types of scenarios and fail to describe other types of experimental protocols. Although they provide models to store data and metadata, these models are very extensible, making the subsequent generation of queries to track the provenance information in the experiment more difficult. This information is frequently written in a non-understandable form, hindering its interpretation by other experimenters who cannot therefore later reproduce or verify the findings (Ruiz-Olazar et al., 2016). The users of software packages that are part of the second group require technical knowledge to write scripts in Python to define the experimental protocols and later execute them. Neuroscience labs need tools with as wide a range of experimental designs as possible that assist the experimenter in the management of all steps of the neuroscience experiments, without being required to have knowledge of a variety of software and programming languages to be able to use them.

### 2.3. The NES Data Model and Main Functionalities

**Figure 1:**
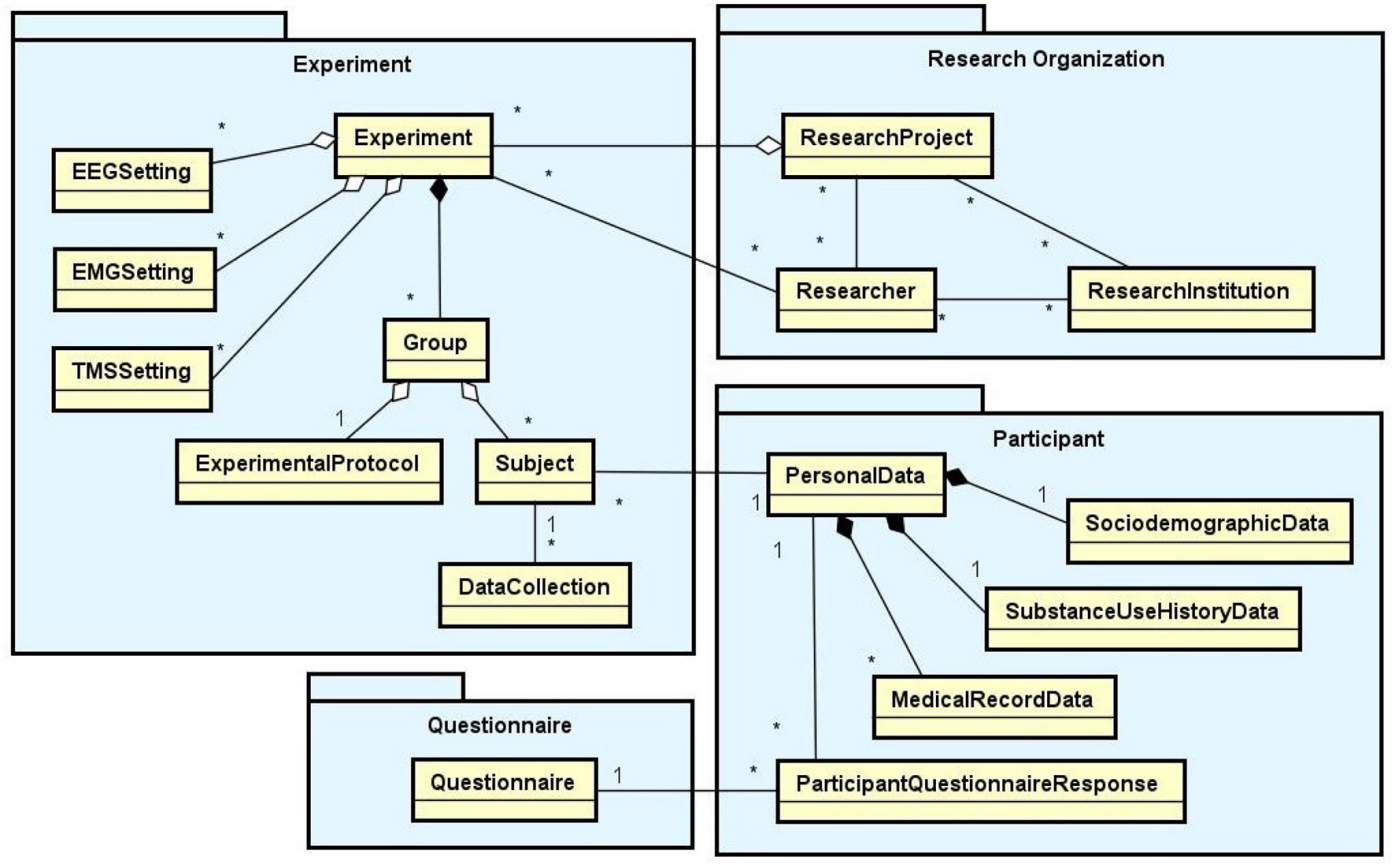
Main data modules of NES (Experiment, Research Organization, Participant, and Questionnaire) with their entity types and relationships. In the diagram, the rectangles represent entity types, the lines ending with black diamonds represent composition relationships, the lines ending with white diamonds represent aggregation relationships, and the conventional lines represent association relationships. The “1” and “*” (that means “many”) near the lines indicate the cardinalities of the relationships. For example: a Group aggregates one ExperimentalProtocol and many Subjects; an Experiment is composed of one or more Groups; a Subject can have many DataCollections, but a DataCollection is associated with only one Subject.

Based on an exhaustive literature research and interviews with domain specialists, we have identified the requirements a software tool should satisfy to support data management in the daily lab routine. In this section, we present the set of functionalities implemented in NES to meet these requirements and the data model that support them. These functionalities provide a complete interface for the storage and management of data and metadata from all the steps of a neuroscience experimental protocol. They are related to a set of database modules represented in the diagram in Figure 1: Experiment, Research Organization, Participant, and Questionnaire. The diagram shows the main entity types of each module and the relationships between them.

According to the NES data model, a research project can have one or more experiments. Each experiment is composed of equipment configurations and one or more groups of subjects, i.e., the individuals that take part in the experiment. Each group can have its own experimental protocol, which is composed of a set of steps. Moreover, each item of data collected in an experiment is associated with a specific step executed in the experimental protocol and the subjects who took part in it. For this reason, to be able to start storing the primary data collected in an experiment in NES, the researcher first needs to register in detail each step involved in the experimental protocol (e.g., the specific preparation for the realization of the experiment).

The NES database modules were designed to store the kinds of data whose structure is common to all experiments, i.e., data that can be described in terms of a standardized structure defined by a database schema. The data model used in NES is aligned with several formats used in neuroscience, enabling interoperability with the most promising initiatives for standardization of data representation for electrophysiology, as much as with guidelines to report neuroscience experiments (e.g., MINI Gibson et al., 2008, MINEMO Frishkoff et al., 2011, and fMRI Poldrack et al., 2008). NES is able to manage several types of electrophysiological data and metadata used by the neuroscience community.

**Figure 2:**
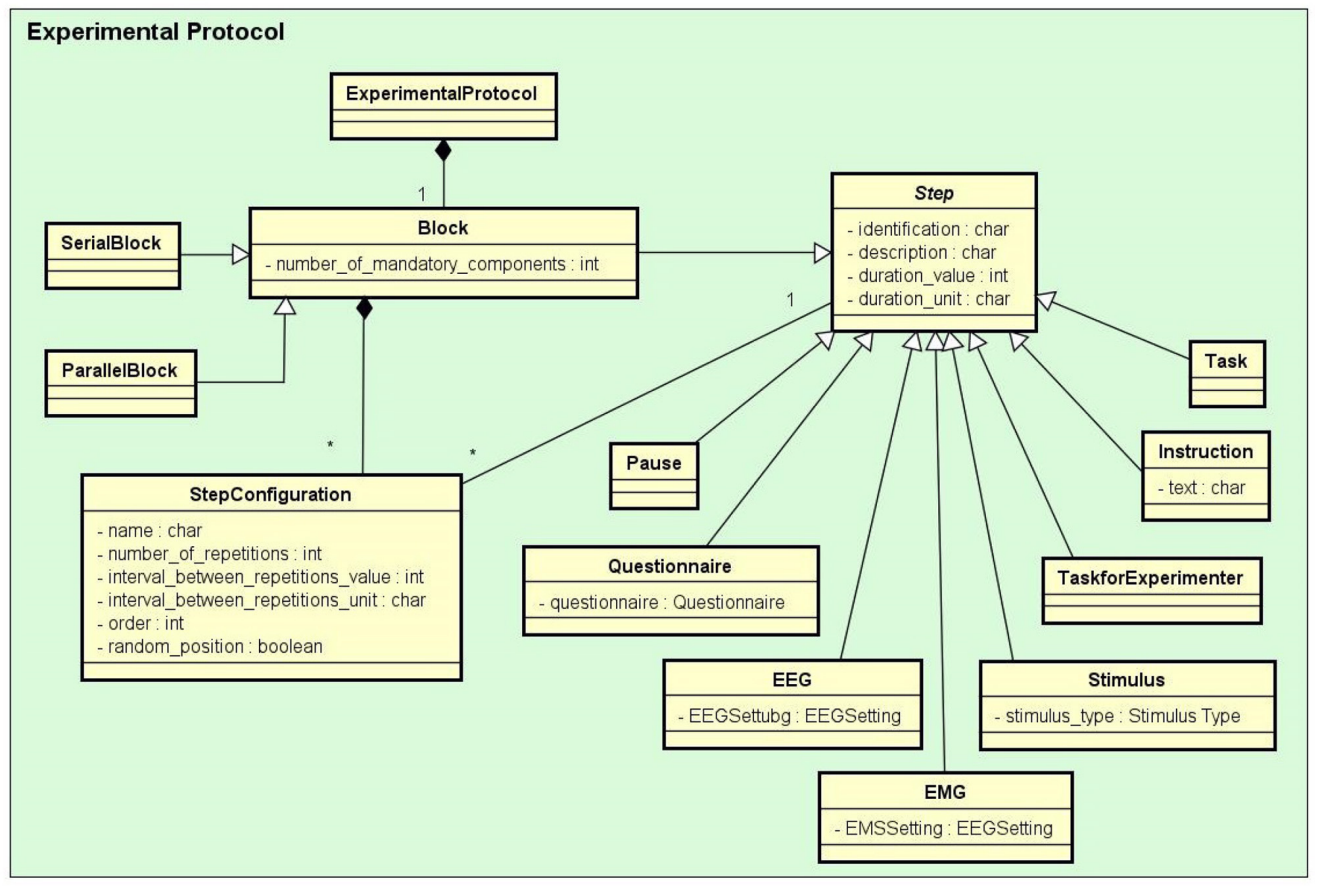
The Experimental Protocol conceptual data schema. In the diagram, the rectangles represent entity types and some of their main attributes, the lines ending with black diamonds represent composition relationships, and the lines ending with triangles represent inheritance relationships. For example: Stimulus, Task, Questionnaire, and EEG are subtypes of Step, each one of them inherits the properties of Step. An ExperimentalProtocol is composed of a Block (of blocks) of StepConfigurations which define how the protocol Steps are performed.

**Figure 3:**
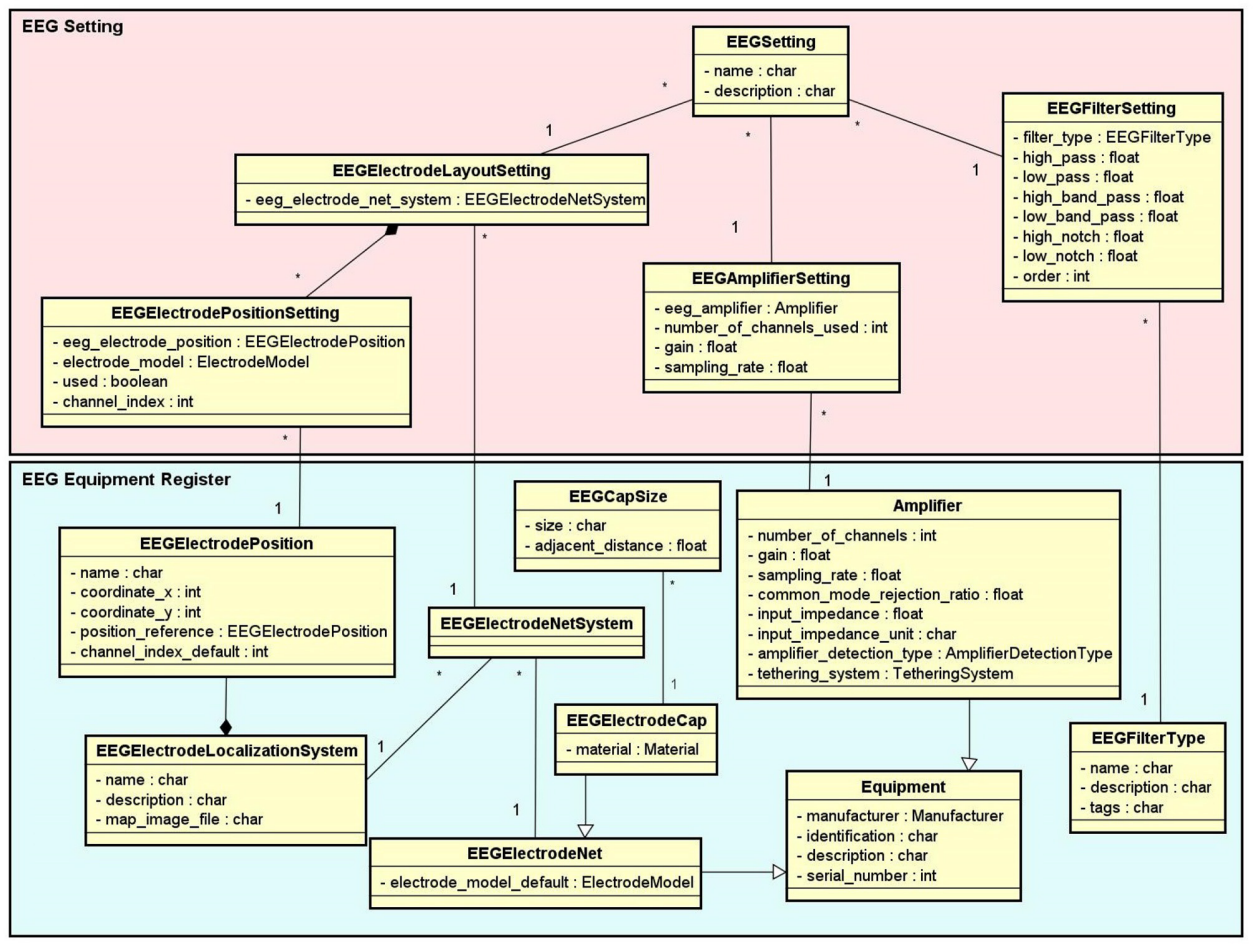
EEG Setting and Equipment Register conceptual data schemas. The EEG Equipment Register schema contains the entity types with the technical characterization associated to a given EEG setup, such as amplifier, filter, electrode net, electrode cap, electrode localization system, etc. The EEG Setting schema contains the entity types which represent the configurations of the EEG equipment used in each step of an EEG experiment.

**Figure 4:**
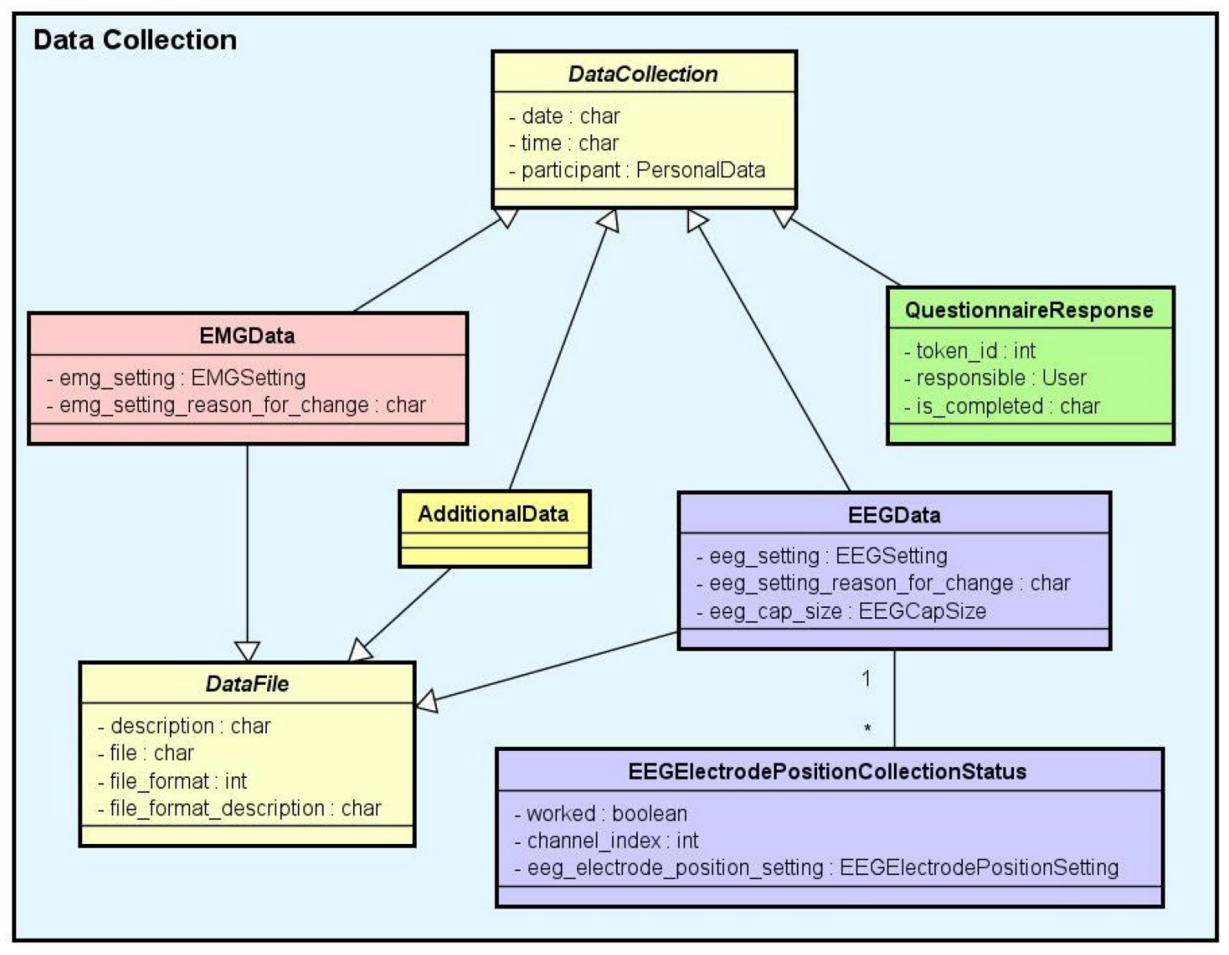
Data collection conceptual data schema. There are several subtypes of data collection: EEG data, EMG data, questionnaire responses, and additional data (for other unlisted data types). Except for the questionnaire responses, all the data collections are files uploaded to the system.

In the following sections, we provide more details about the database schema of each module. Figures 1–**4** are conceptual database schemas expressed using UML Class Diagrams. They purposely abstract details about the real database structure in order to make the diagrams easier to read and understand.

#### 2.3.1. Participant Module

The Participant module supports functionalities to manage information related to the participants in the experiments. Its data schema specifies attributes of the participants that are significant for the experiments' design and interpretation.

In the Participant data schema, as can be seen in Figure 1, the participant data is divided in five components:

- *Personal data* contains participants' basic information—identification, name, gender, birth date, address, and phone numbers.
- *Social Demographic data* registers the participants' native country, occupation, religion, and race.
- *Substance Use History data* allows to register participants' history of use of alcohol, tobacco, and other drugs.
- *Medical Evaluation* allows storage of participants' medical records (including diagnosis with ICD codes and medical tests).
- *Questionnaire Response* includes the participants' answers to the questionnaires that are part of experimental protocols.

#### 2.3.2. Experiment Module

The Experiment module is the core of NES. It supports functionalities for experiment registration and configuration as well as data collection. Its data schema was designed to be able to represent the structure and data of types of experiments frequently performed in humans, in addition to data stored in other widely used formats in neuroscience without any loss of information. For this purpose, the data schema organizes data related to the experiments, the groups of subjects, the steps of their experimental protocols, and the equipment settings. Each of these types of entities is described in more detail below.

- *Experiment*: It stores information that identifies the experiments and their purposes, the responsible researchers, and the resulting publications.
- *Experimental Protocol*: An experimental protocol is modeled in NES as a workflow composed of blocks of parallel or sequential steps. There are several types of steps that a block may contain (e.g., task, stimuli, TMS, instructions, EEG, EMG, questionnaire administration, and other types of data collection). Each block or step can have its own configuration, such as the number of times it must be repeated, the time interval between repetitions, and its order in the protocol workflow, which can be deterministic or random. Figure 2 shows the conceptual schema of the experimental protocol data.
- *Group*: Several groups of subjects can be created for an experiment. Each group is associated with an experimental protocol and the participants who take part of it, as shown in Figure 1.
- *Equipment Setting*: It stores information about equipment and materials of an eletrophysiology experiment. Each setting is associated with a step in an experimental protocol. NES can store the settings of the equipment used to record raw data, as well as the type of materials used in each data acquisition procedure. For EEG settings, as shown in Figure 3, NES can store amplifier settings and filter settings. The amplifier settings include gain, number of channels, common mode rejection rate, input impedance, and unit of impedance, among other information. The filter settings include information about the filter type, high pass cutoff, low pass cutoff, and order.
  Another important item of information considered in the Equipment Setting data schema is the electrode layout. NES allows electrode settings to be recorded individually or using an electrode net system, as the 10–20 system^2^. It also allows the registration of the electrode model, the electrode positions and their channel index. The spatial coordinates of each electrode, its position reference, and its default channel index can also be registered.
  For EMG experiments, in addition to storing information about the equipment and materials, NES can store systems for electrode placement, such as the SENIAM system (Surface ElectroMyoGraphy for Non-invasive Assessment of Muscles). Additionally, a list of muscles and its subdivision where the electrodes will be located can be recorded.
  For the Transcranial Magnetic Stimulation (TMS) setting, NES can store data about the TMS device and the coil model used in the experiment.
- *Data Collection*: This is another key component of the Experiment module. It supports the functionalities related to the management of data collected during the execution of the experimental protocol steps. NES attaches the data collection for each step of an experiment to the subject who took part in it. As shown in the conceptual schema of Figure 4, NES is able to handle several types of data collections: raw data obtained from a signal acquisition equipment (e.g., EEG and EMG), questionnaire responses, and any other type of additional files (such as spreadsheets, videos, and textual notes) that can be collected or generated in an experiment. The possibility of uploading additional files is also useful to store processed data alongside the raw data collected during the experiment. For example, a researcher can add one or more data collection steps at the end of the protocol of an experiment specifically to register the data (files) derived from the raw data collected in the previous steps. Information about how the derived data was produced can be registered in the form of textual notes.
  The data model allows information about the file type format, and the date and time of the acquisition to be stored. In the case of EEG data acquisition, it is possible to record, for each participant, the size of the electrode cap and information related to electrode positions and their status (e.g., used or not used) at the moment of the capture. The settings of the equipment and materials used in each data acquisition procedure can also be stored. This information is fundamental to enable the sharing and reuse of the raw data.

In NES, it is possible to create copies of an experiment (with or without the associated data collections). The copies are fully accessible through the user interface. This is useful for versioning control. One can also export an experiment and generate a .zip file with all the experiments' data and metadata. The .zip file can be later imported into other studies or NES installations. Other details about the export features are provided in section 2.3.5.

#### 2.3.3. Questionnaire Module

Questionnaires are a very flexible way to collect data from study participants. In NES, a questionnaire can be configured as a step of an experimental protocol.

As questionnaires vary from study to study, they are difficult to be stored in a rigid, fixed database structure. To conveniently deal with this problem and also to provide more quality and security to data collected through questionnaires, a questionnaire management system, the LimeSurvey^3^, was integrated into NES. This kind of system is a powerful, easy-to-use tool to create electronic question-and-answer surveys.

#### 2.3.4. Research Organization Module

The Research Organization module supports functionalities to register information of the researchers that are working on the studies, their projects and the institutions involved. Its data schema stores data about the researchers, laboratories, and projects associated with the experiments. NES allows multiple experiments within the same research project, where each experiment can involve a different group of researchers. Researchers participating in an experiment have their own access and permissions to manage the experiment in NES.

NES implements a role-based control access (RBAC) approach to restrict system access to authorized users. RBAC is defined around roles (of users) and permissions (to perform particular system functions). Multiple permissions can be granted to a role, and a user can have multiple roles. Thus, it is simple to manage permissions since users can be grouped according to their roles. NES has some default groups of users (such as Administrator, Junior Researcher, and Senior Researcher). However, using the Administration Interface, one may add or change roles and the permissions for each role to best suit the needs of the research institution. Examples of permissions which can be attributed to groups are: “Can view research project,” “Can add study,” “Can change subject,” “Can delete survey,” and “Can export experiment.”

#### 2.3.5. Data Export Functionality

An important functionality provided by NES which is connected with all its data modules is the export of experimental data and metadata. This functionality includes the data from participants (e.g., clinical diagnoses, socio-demographics), the data collected in the experiment execution (e.g., questionnaire responses, electrophysiological raw data), and metadata about the experimental protocol (e.g., description of the purpose of the experiment, description of the protocol steps, equipment configuration, and notes made by researchers).

Through the NES export functionality, a researcher is able to download experimental data and metadata in interoperable formats. It was implemented within the Frictionless Data philosophical and technical framework in order to decrease friction that is commonly associated with understanding data and metadata (Peschanski et al., 2020). Frictionless Data is an open-source framework for building data infrastructure. It was established by the Open Knowledge Foundation^4^ to provide technical support to open science strategies. The framework includes various data standards to help to describe data. Its core specification, the Data Package, is a container format used for storing metadata alongside a dataset expressed as a simple JSON file named datapackage.json (Fowler et al., 2017).

NES offers two types of file organization in an export: per participant and per experiment. In both types, one can filter the group of participants whose data will be exported. The participants can be filtered, for example, by gender, location, diagnosis, and age. One can also select the participant data fields to be included in the export. For experiments with questionnaires, it is possible to choose the questions to be included.

**Figure 5:**
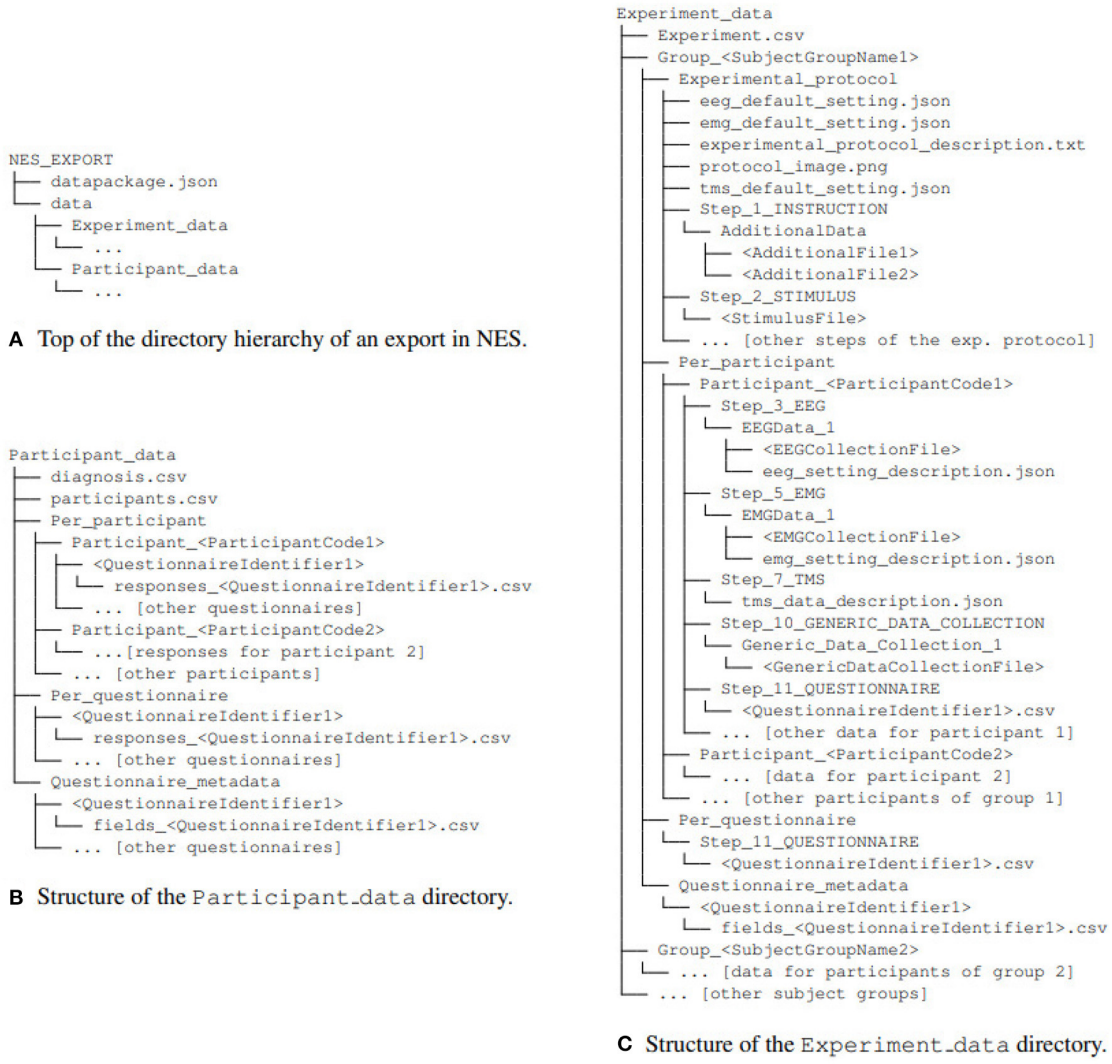
Directory structure of an experiment dataset exported from NES (adapted from Peschanski et al., 2020). The root directory **(A)**, NEX_EXPORT, contains the directory data, with the data package files, and the file datapackage.json, which describes the structure and contents of the data package (as prescribed by the Frictionless Data standard). The experiment data is organized in two directories: Participant_data **(B)** and Experiment_data **(C)**. The former contains the data from the participants (including their responses for questionnaires applied outside the context of experiments), while the latter contains all data collected within the steps of the experimental protocol (and their metadata).

Figure 5 shows the directory structure of an experiment dataset exported from NES. The structured data is exported in plain-text files in the CSV (*comma separated values*), containing both textual and numeric data. The equipment configuration is exported in JSON format. The EEG raw data can also be exported in the Neurodata Without Border (NWB 1) format (Teeters et al., 2015), a prominent initiative for standardization of data representation for neurophysiology. An NWB 1 file consists of several main groups, each of which is a container (similar to a directory) for different subsets of the data. In NES, the data included in the NWB 1 file is organized in three main groups: *General metadata, Device configuration*, and *Data acquisition*. *General metadata* contains information about the description of the experiment and some demographics data of the participants [e.g., experiment, subject id, sex, genotype (flesh tone), subject (natural of)]. *Device configuration* group contains the settings of the devices used in the EEG data collection (e.g., amplifier, filter device, electrode net layout). The *Data acquisition* group contains the raw data and metadata collected for each session of EEG (e.g., data, time, number of samples, electrode indexes, number of channels).

## 3. Results

### 3.1. The Developed Software System

**Figure 6:**
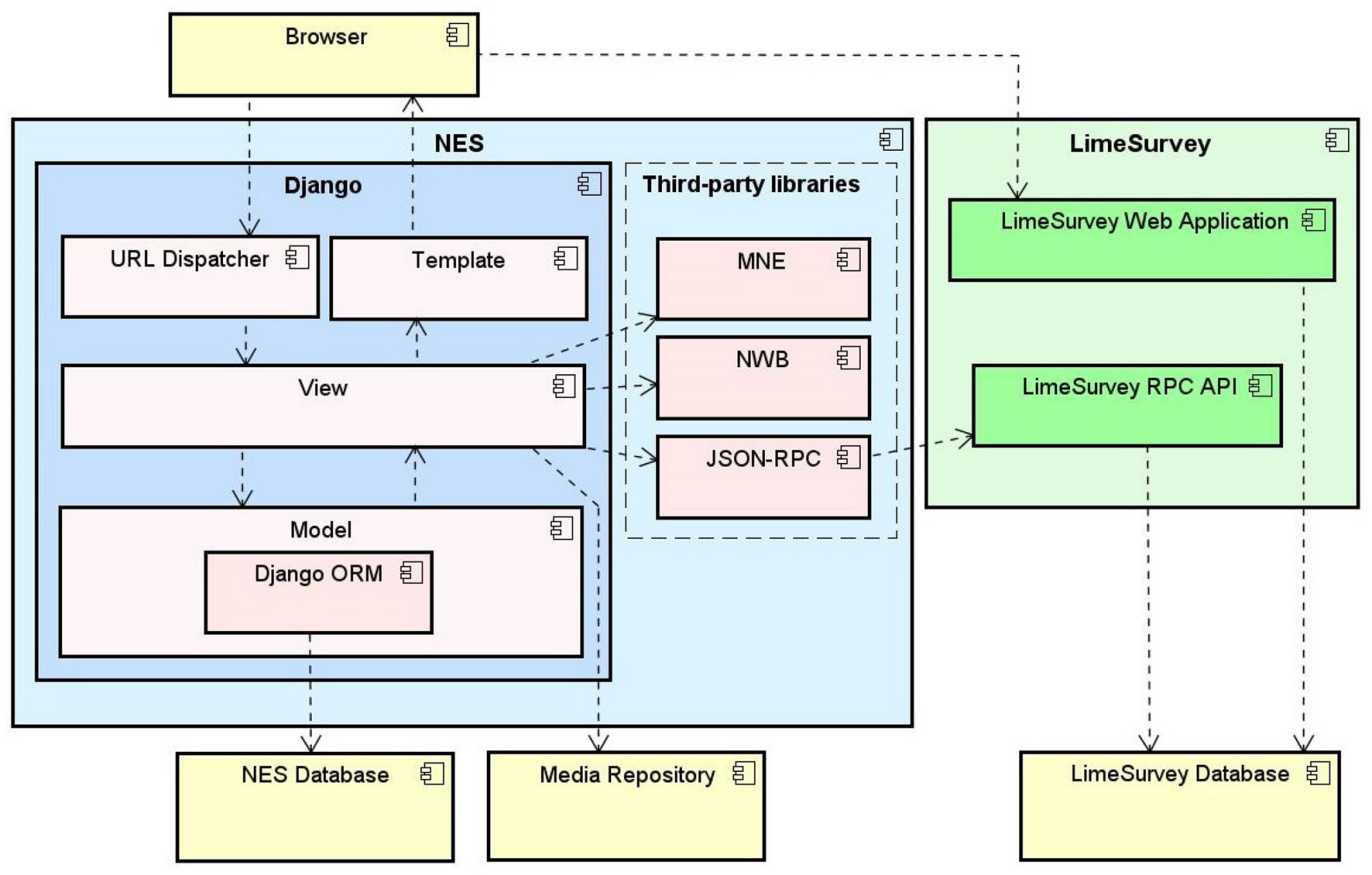
NES software architecture, composed by the Django Web framework components and third-party libraries to convert data and provide interoperability with other tools.

The functionalities and the data model described in section 2.3 were implemented in a platform-independent Web system, whose architecture is depicted at a high level in Figure 6. It is a standard three-tier Web architecture, with a data storage tier, an application layer, and a presentation tier.

The data storage tier is implemented as a relational database in the open-source database management system PostgreSQL^5^. The database is used to store all the structured data, according to the models described in section 2.3. Physical files, such as the EEG and EMG recordings and user additional data, are stored in the file-server system.

The application layer consists of a group of libraries that perform the execution engine of the system in the server side. NES was implemented in Python, using the Django Web framework^6^. This platform offers many benefits, such as scientific libraries, extensive documentation, and an active community. Python is an open-source programming language that has become one of the most popular programming language used in neuroscience systems.

NES uses third party libraries (e.g., MNE^7^, NWB^8^ and JSON-RPC^9^) to provide interoperability with other neuroscience tools. The MNE is an open-source Python software for exploring, visualizing, and analyzing human neurophysiological data such as Magnetoencephalography (MEG), EEG, sEEG and more. NES uses MNE to read and visualize several raw EEG data formats. The NWB-API is a Python API that NES uses to create NWB 1 files.

NES integrates with LimeSurvey to support the use of electronic questionnaires to collect data in experiments. LimeSurvey is an open-source, Web server-based software. It enables the storage of all data collected through the questionnaires in a “private” server. It relies on an underlying database management software which can be deployed on a server that is deemed appropriate to store the target data and customized to support different data access policies. With this structure, NES has full control over LimeSurvey data storage and access. NES communicates with the LimeSurvey application through the RemoteControl 2 API^10^. This API is a XML-RPC/JSON-RPC based Web service which offers several functions for questionnaire management.

Users access NES via a browser. The presentation tier is implemented using the Twitter Bootstrap^11^ framework to generate the application layout and make it responsive, adjusting the Web pages dynamically according to the device used (e.g., desktop, mobile, tablet). Additionally, JavaScript was used to facilitate the implementation of some functionalities.

NES is an internationalized software, it can be adapted to various languages and regions without engineering changes. Currently, NES is localized to Brazilian Portuguese (pt-br) and English (en), but it can be localized to other languages by simply translating text and adding locale-specific components.

**Figure 7:**
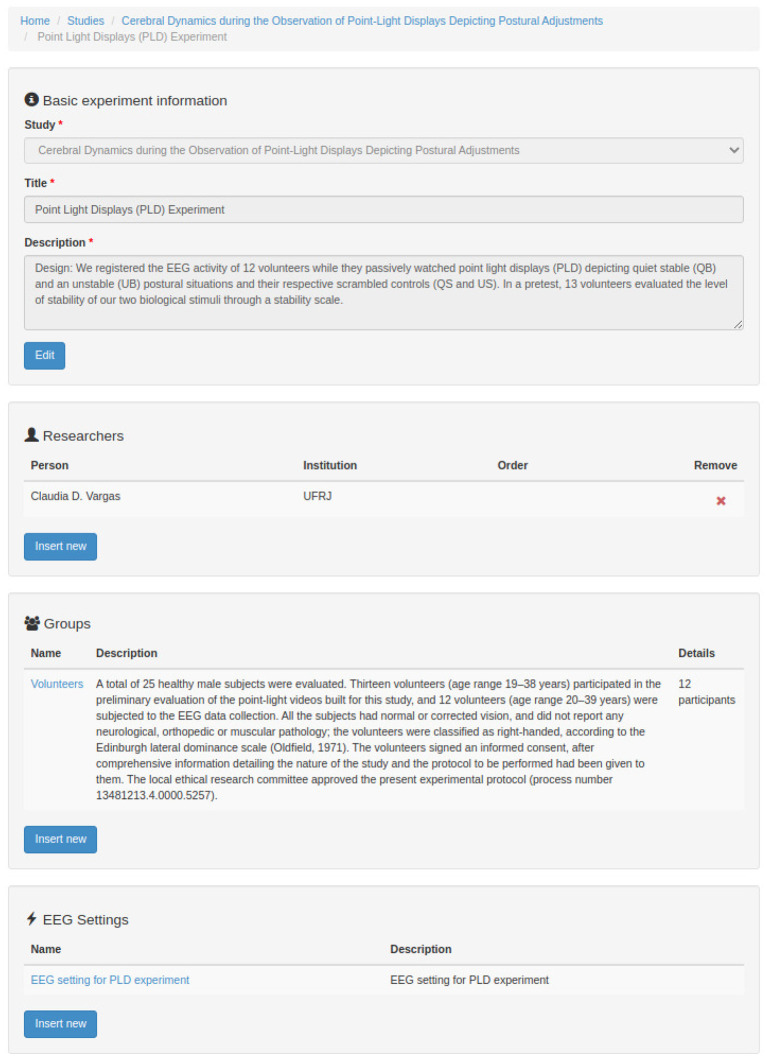
NES graphical interface for registering the basic information about an experiment.

**Figure 8:**
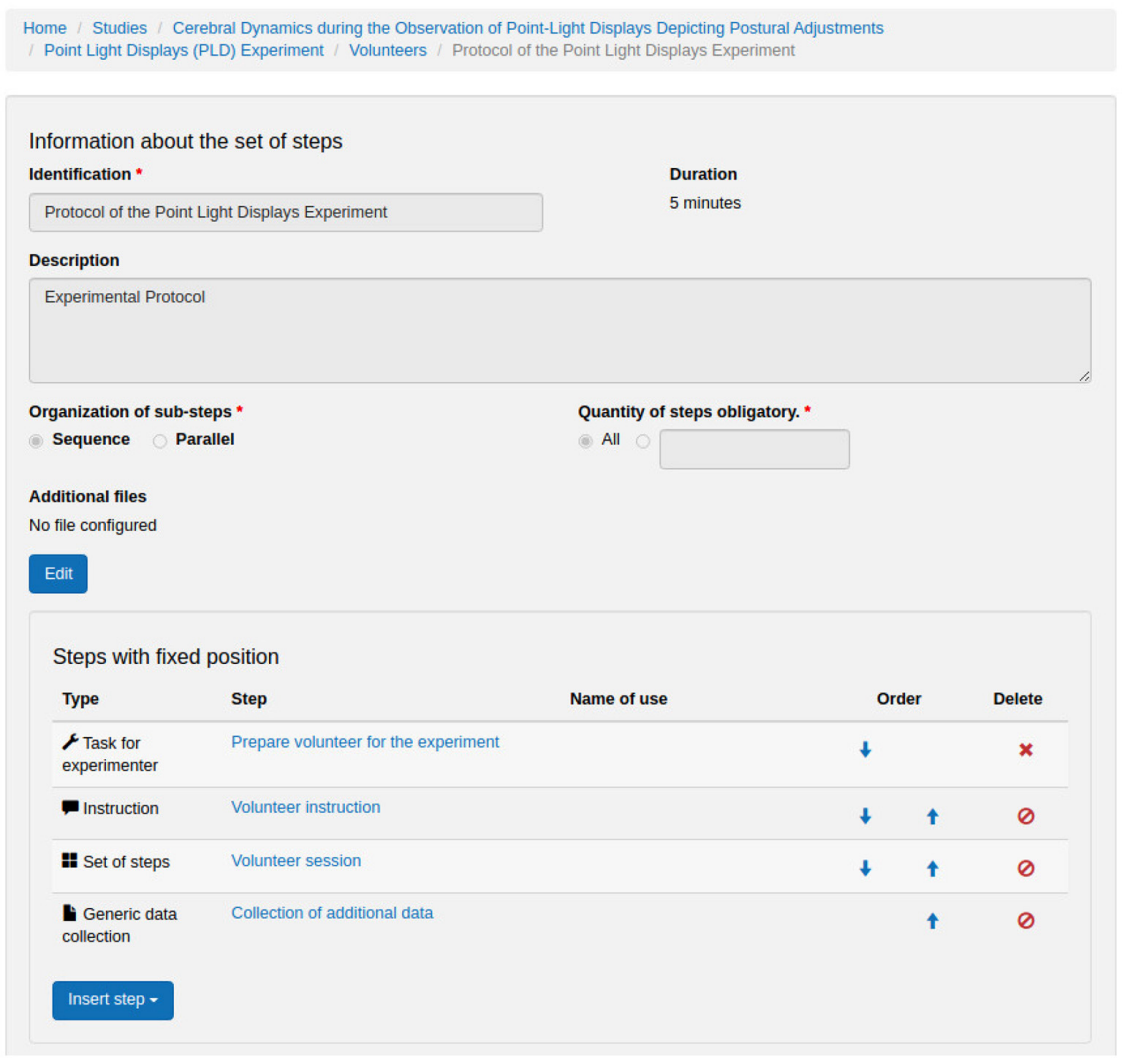
NES graphical interface for registering an experimental protocol for a group of participants.

**Figure 9:**
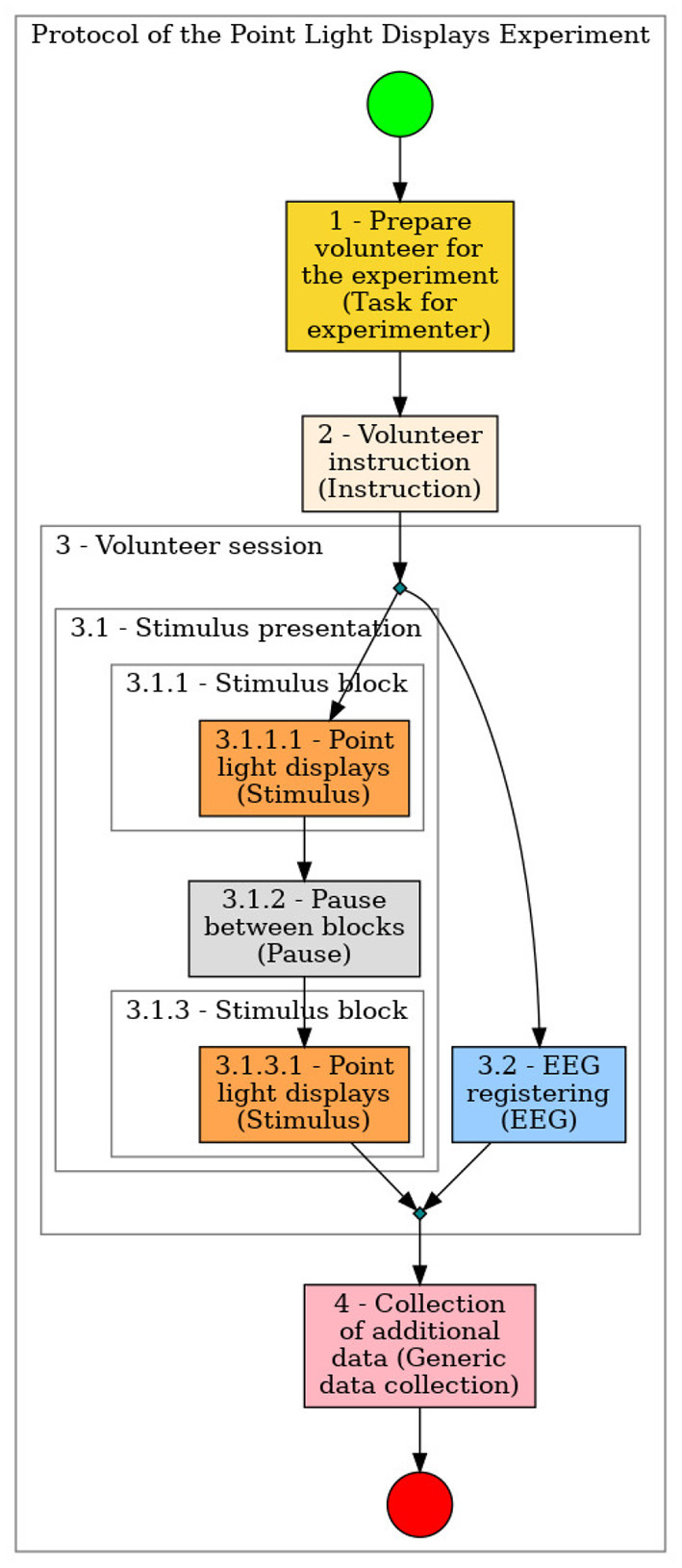
Example of diagram generated by NES from an experimental protocol definition.

**Figure 10:**
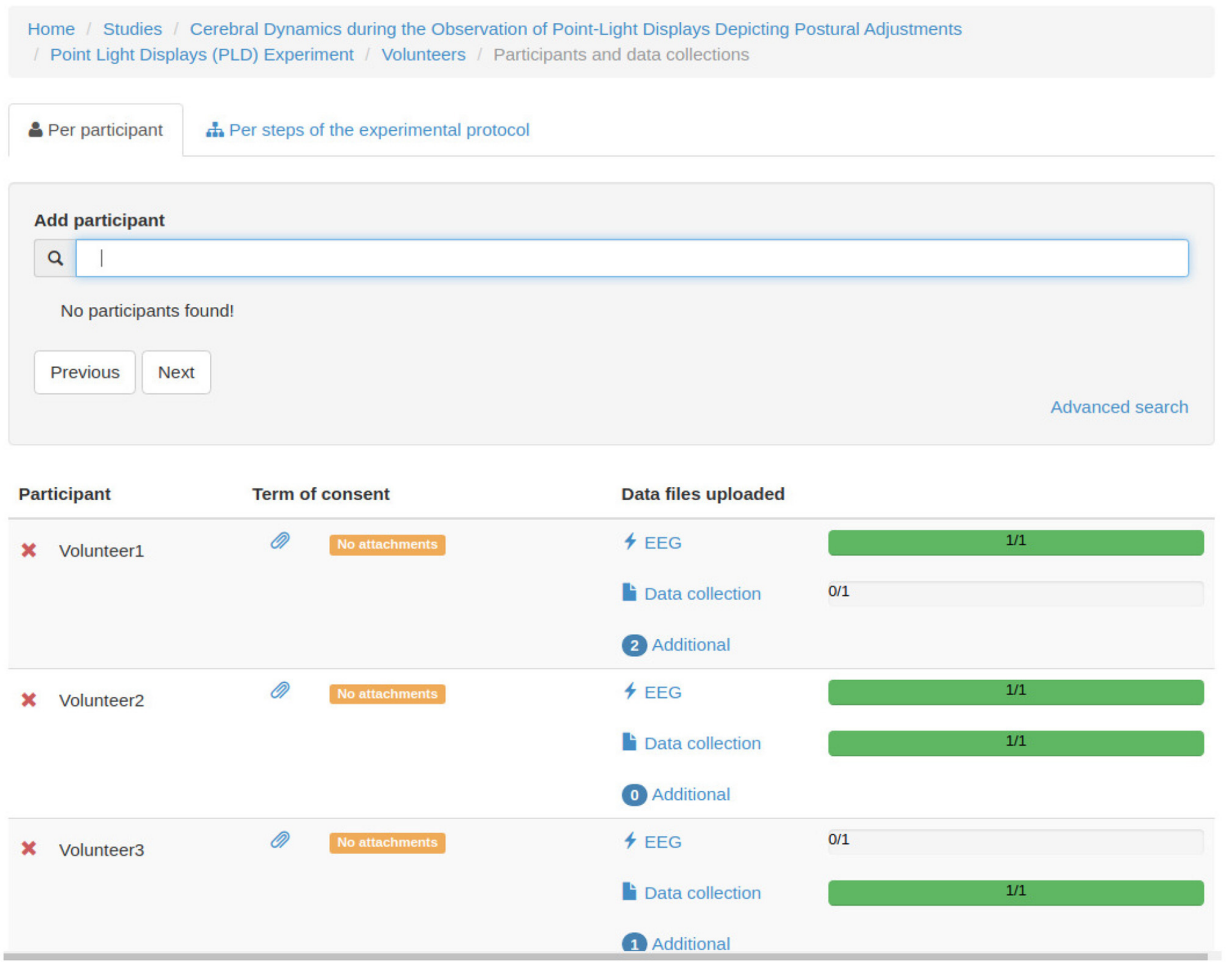
NES graphical interface for listing participants and data collections of an experiment.

**Figure 11:**
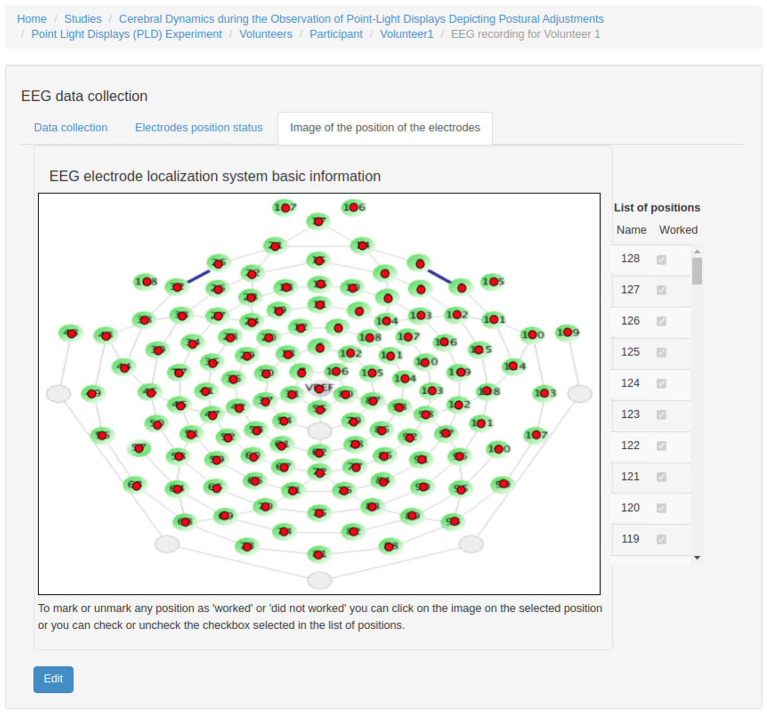
NES graphical interface for registering electrode status in EEG data collection.

**Figure 12:**
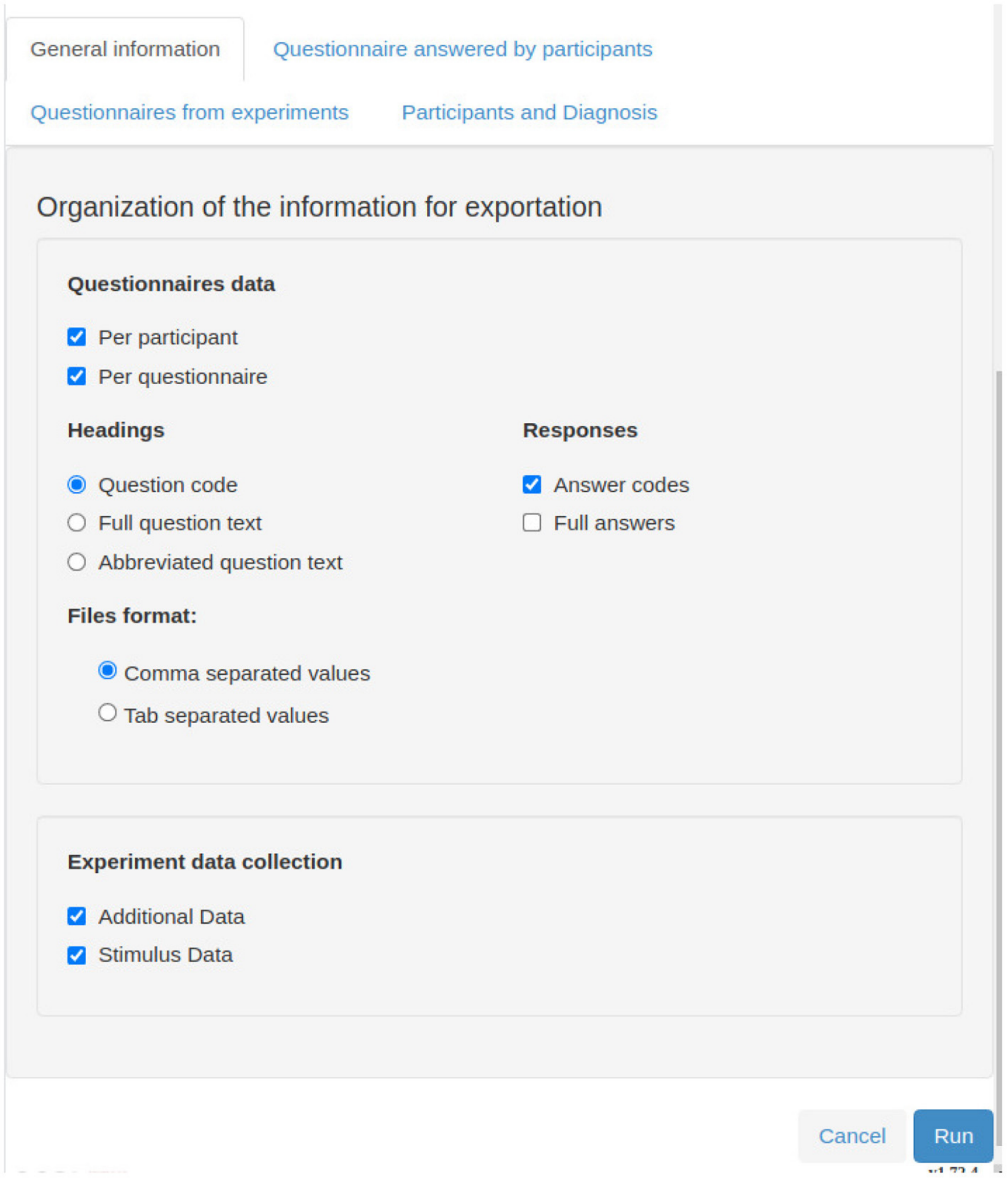
NES graphical interface for selecting data export options.

Figures 7–**12** show some screenshots from the NES Web interface. Figure 7 presents the NES page for registering the basic information about an experiment. It enables users to include groups of subjects for the experiment and equipment settings for the electrophysiology data captures. Figure 8 shows the page for the definition of the experimental protocol of a group of subjects of an experiment. The experimental protocol is described as a workflow, which can contain blocks of sequential or parallel steps of several types. An example of workflow diagram NES automatically generates from a protocol definition is shown in Figure 9. In the protocol illustrated by the diagram, the *Stimulus presentation* and the *EEG registering* are, respectively, a block of stimulus steps and a data collection step which are performed in parallel in the experiment, according to the protocol. Figure 10 shows the page for listing participants and data collections of an experiment. In Figure 11, the NES interface shows the position of the electrodes used in an EEG data collection. The interface allows the working electrodes used in the EEG recording to be indicated. Finally, Figure 12 shows some configurations a user can set in the export of experimental data and metadata.

NES is licensed under the Mozilla Public License version 2.0 and its source code and documentation are available at https://github.com/neuromat/nes. The online software documentation and User Guide, with comprehensive descriptions of the NES functionalities, are available at https://nes.readthedocs.io/en/latest/.

### 3.2. Using NES to Create Open Databases

**Figure 13:**
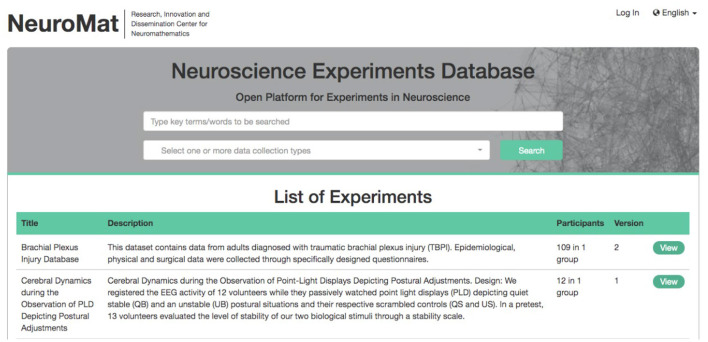
NeuroMat Open Database (NeuroMat DB) home page.

NES is a software that can be installed by a laboratory or a research group to locally manage experiments and their data. Besides, NES can also be used to support the generation of well-documented, anonymized datasets that can be published to openly share experimental data. An example of such an application can be seen in the NeuroMat Open Database (NeuroMat DB)^12^, an initiative that provides an open-access platform for sharing and searching data and metadata from neuroscience experiments. Electroencephalographic (Martins et al., 2017; Hernández et al., 2021) and clinical data (Patroclo et al., 2019; Ramalho et al., 2019) collected within NeuroMat and organized in NES were made publicly available through the NeuroMat DB Web portal. Figure 13 shows a page that lists these datasets.

**Figure 14:**
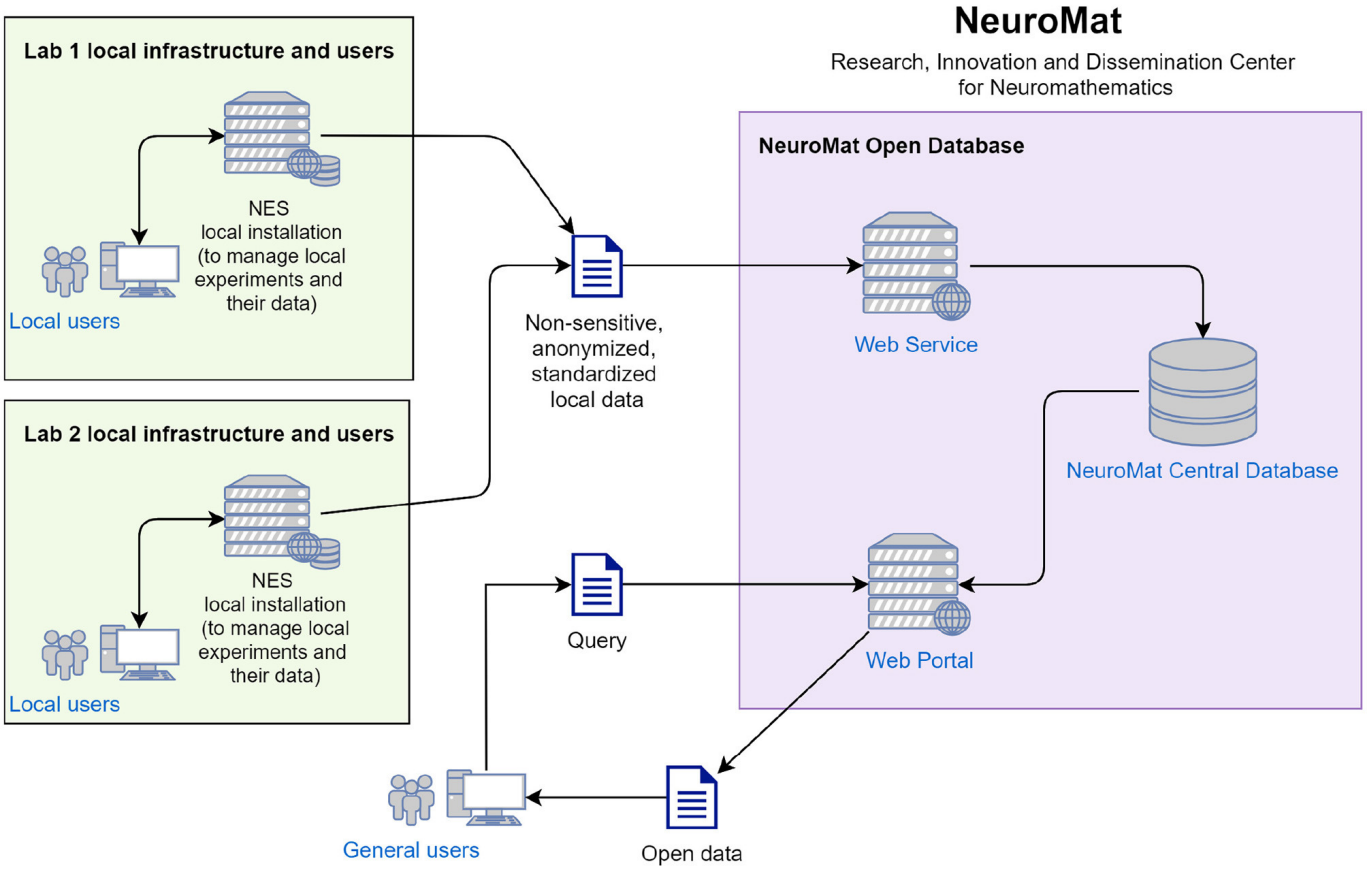
Data capture with NES and public sharing in the NeuroMat Open Database.

Through NES, a researcher is able to send data and metadata of his/her experiments to the Open Database (as illustrated in Figure 14). Before sending the data, NES replaces the experiment participant's identifiers with random numbers in order to anonymize them. Personally identifiable information such as name, document number, address, and phone number can not be sent. In principle, the researcher is responsible for choosing among the study data what is going to be sent to the portal. All data is cryptographed upon transmission to the Open Database. When a new dataset arrives at the Open Database, it is evaluated by a curatorial committee who decides whether it is appropriate for publication. The committee then guarantees that no sensitive information will be made publicly available in the open database. If approved by the committee, the dataset is published on the portal.

#### 3.2.1. The Brachial Plexus Injury Database: A Case Study

The Center for Research in Neuroscience and Rehabilitation (NPNR) of the Deolindo Couto Institute of Neurology (INDC) at the Federal University of Rio de Janeiro (UFRJ), in collaboration with NeuroMat, investigated the brain plasticity that follows traumatic brachial plexus injury (TBPI) and its surgical reconstruction. The brachial plexus is composed of a set of peripheral nerves responsible for the sensory, motor, and autonomic innervation of the upper limbs. Injury to peripheral nerve structures and/or medullary avulsion as a result of a TBPI lead to changes in cortical representations and are also often associated with neuropathic pain (Torres et al., 2019). In recent years, the frequency of this type of injury (mainly caused by motorcycles accidents) has grown considerably in developing countries and has already become a public health concern. NPNR is using NES to collect, store and manage data from the TBPI studies, which are mainly made up of electrophysiological recording, responses to clinical questionnaires, and behavioral data from more than 170 patients. An anonymized portion from the TBPI database on NES was made publicly available on the NeuroMat Open Database Web portal^13^. As far as we know, this is the first worldwide open digital database centered on adult TBPI (Patroclo et al., 2019).

## 4. Discussion

We identified the guidelines and models most widely used by neuroscientists in the representation and storage of experimental data (Ruiz-Olazar et al., 2016) and incorporated them in NES. To the best of our knowledge, there are no other open-source software tools which provide facilities to record the data and metadata involved in all steps of a neuroscience experiment.

NES provides a structured and comprehensive platform with robust tracking of data provenance that is fundamental to enable the reproduction of the experiment. It was developed to keep together experimental data and information on its provenance, defined by the seven W's (Who, What, Where, Why, When, Which, (W) how). Examples of provenance information maintained by NES are: information about the scientists responsible for the experiment and collection of data and the description of the subject groups (who); the details about the recording protocol or behavioral data collection (e.g., the types of data collection performed) (what); the details of the experimental protocol used in the collection of primary data (how); the start/end date-time for data collection (when); the purpose of the experiment (why); the information about the experimental conditions to which the groups of subjects are submitted, such as tasks performed and stimuli applied (which); the information about the laboratory where data was collected (where) and even publications or other results that have arisen from the study of the collected data. Scientists can also record additional details for each participant in the experiment, such as information about his/her clinical history and social-demographic data.

It is worth mentioning that NES is not a new way to standardize the representation of experimental data. There are several models and formats (e.g., NeoHDF5 Garcia et al., 2014, NWB Teeters et al., 2015, and NIX Stoewer et al., 2014) currently in development to address this issue. These models are appropriate for organizing and exchanging data of a particular type and from a particular experiment. However, they do not replace the function of a database system, as provided by NES.

A database system keeps large data volumes and provides functionalities for access control, data consistency, fault tolerance and efficient data recovery. Furthermore, in a database it is possible to store the relationships between different types of data from different experiments, allowing for more sophisticated data analysis which are especially valuable to support research in multidisciplinary domains.

The NES Web interface and modular format provide an intuitive use of its data management functionalities and do not depend on any specific knowledge on informatics. NES was developed using open technologies and tools— such as the Django web framework and the PostgreSQL database management system—which can be easily installed and used in any research laboratory. Moreover, these tools make it capable of supporting a large number of simultaneous users and handling large amounts of data. All the structured data managed by NES is stored in PostgreSQL. This includes the participant records, the description of the experimental protocols, the equipment settings, the research organization data, among others. This kind of data is efficiently handled with PostgreSQL.

All the files uploaded by users in NES, e.g., the EEG and EMG recordings and other types of data collections, are stored in the file-server system to facilitate their manipulation. This approach also enables the use of a distributed file system, a network storage device, or a cloud file storage to have storage scalability in a transparent way for NES.

NES is being used to manage experimental data of studies conducted in the NeuroMat research center. In particular, it has been used to construct an open database with data from TBPI studies. This initiative may allow the identification of functional markers related to the patients clinical improvement and foster the development of new investigative tools to unveil its mechanisms. Moreover, it aims at reducing the distance between clinical and experimental practice and encourage data sharing and reuse.

### 4.1. Limitations and Future Directions

NES provides basic functionalities for the registration of medical records because these data are required in several kinds of neuroscience experiments. However, it is important to emphasize that NES is not an electronic data capture (EDC) nor an electronic medical record (EMR) system and was not designed for these purposes. NES is not able to register information of nonhuman subjects. But the Participant data model can be easily extended to accommodate this type of data. Examples of attributes that should be considered for nonhuman subjects are identification, age, species, sex, stock or strain, house, and genetic characterization.

NES has some simple features for data search and filtering. For instance, one can search for a given participant in a given study and perform basic filtering in the exportation module. Also, data filtering can be employed upon data transference to the NeuroMat Open Database (NeuroMat DB) portal. The portal supports data search using keywords via an Elasticsearch mechanism. To improve the tool with more advanced querying features, a new module for Data Searching and Visualization which will index all the data stored in the NES database is being designed.

NES has special functionalities that facilitate the management of electrophysiological data and metadata (i.e., EEG and EMG). However, currently it has limited support for experiments involving neuroimaging. For example, NES can read several EEG data formats and extract metadata from them, but it does not have an equivalent functionality to handle MRI and fMRI data. The extension of NES with a neuroimaging module will be implemented in the context of a scientific collaboration recently established with researchers from the Polytechnic Faculty of the National University of Asunción. In order to provide scalable storage for neuroimages, NES will be transformed into a cloud native system. This cooperative project also foresees the deploying of NES in research laboratories of the Neurology service of the Central Hospital of the Social Security Institute, in Asunción, Paraguay, to support studies of neurological disorders.

## Author Contributions

KB and CV conceived the original concept for the software. MR-O and ES contributed to the software implementation. MR-O and KB wrote the first draft of the manuscript. ER drew the conceptual data schema diagrams. All authors contributed to the data model, software design, revised the manuscript, and approved the submitted version.

## Funding

This work was supported from research activity conducted as part of the Research, Innovation and Dissemination Center for Neuromathematics (NeuroMat) and funded by the São Paulo Research Foundation-FAPESP (No. 2013/07699-0), Brazilian National Council for Scientific and Technological Development-CNPq (No. 426579/2016-0 and 309560/2017-9), Fundação de Amparo à Pesquisa do Estado do Rio de Janeiro-FAPERJ (No. E26/010002474/2016, CNE 202.785/2018, and E-26/010.002418/2019), Financiadora de Estudos e Projetos-FINEP (PROINFRA HOSPITALAR No. 18.569-8), and Paraguayan National Council of Science and Technology-CONACYT with support from FEEI (No. PINV18-665).

## Conflict of Interest

The authors declare that the research was conducted in the absence of any commercial or financial relationships that could be construed as a potential conflict of interest.

## Publisher's Note

All claims expressed in this article are solely those of the authors and do not necessarily represent those of their affiliated organizations, or those of the publisher, the editors and the reviewers. Any product that may be evaluated in this article, or claim that may be made by its manufacturer, is not guaranteed or endorsed by the publisher.

## Footnotes

^1^ https://neuromat.numec.prp.usp.br/

^2^The 10–20 system is an internationally recognized method to describe and apply the location of scalp electrodes in EEG experiments.

^3^ https://www.limesurvey.org/

^4^ https://okfn.org/

^5^ https://www.postgresql.org/

^6^ https://www.djangoproject.com/

^7^ https://mne.tools/

^8^ https://pynwb.readthedocs.io/

^9^ https://www.jsonrpc.org/

^10^ https://manual.limesurvey.org/RemoteControl_2_API

^11^ https://getbootstrap.com/

^12^ http://neuromatdb.numec.prp.usp.br/

^13^ https://neuromatdb.numec.prp.usp.br/experiments/brachial-plexus-injury-database/
